# Comprehensive Analysis of PPARα-Dependent Regulation of Hepatic Lipid Metabolism by Expression Profiling

**DOI:** 10.1155/2007/26839

**Published:** 2007-09-13

**Authors:** Maryam Rakhshandehroo, Linda M. Sanderson, Merja Matilainen, Rinke Stienstra, Carsten Carlberg, Philip J. de Groot, Michael Müller, Sander Kersten

**Affiliations:** ^1^Nutrigenomics Consortium, Wageningen Centre for Food Sciences, P.O. BOX 557, 6700 AN Wageningen, The Netherlands; ^2^Nutrition, Metabolism and Genomics Group, Division of Human Nutrition, Wageningen University, P.O. BOX 8129, 6700 EV Wageningen, The Netherlands; ^3^Life Sciences Research Unit, University of Luxembourg, 162A Avenue de la Faïencerie, 1511 Luxembourg, Luxembourg

## Abstract

PPARα is a ligand-activated transcription factor involved in the regulation of nutrient metabolism and inflammation. Although much is already known about the function of PPARα in hepatic lipid metabolism, many PPARα-dependent pathways and genes have yet to be discovered. In order to obtain an overview of PPARα-regulated genes relevant to lipid metabolism, and to probe for novel candidate PPARα target genes, livers from several animal studies in which PPARα was activated and/or disabled were analyzed by Affymetrix GeneChips. Numerous novel PPARα-regulated genes relevant to lipid metabolism were identified. Out of this set of genes, eight genes were singled out for study of PPARα-dependent regulation in mouse liver and in mouse, rat, and human primary hepatocytes, including thioredoxin interacting protein (Txnip), electron-transferring-flavoprotein β polypeptide (Etfb), electron-transferring-flavoprotein dehydrogenase (Etfdh), phosphatidylcholine transfer protein (Pctp), endothelial lipase (EL, Lipg), adipose triglyceride lipase (Pnpla2), hormone-sensitive lipase (HSL, Lipe), and monoglyceride lipase (Mgll). Using an in silico screening approach, one or more PPAR response elements (PPREs) were identified in each of these genes. Regulation of Pnpla2, Lipe, and Mgll, which are involved in triglyceride hydrolysis, was studied under conditions of elevated hepatic lipids. In wild-type mice fed a high fat diet, the decrease in hepatic lipids following treatment with the PPARα agonist Wy14643 was paralleled by significant up-regulation of Pnpla2, Lipe, and Mgll, suggesting that induction of triglyceride hydrolysis may contribute to the anti-steatotic role of PPARα. Our study illustrates the power of transcriptional profiling to uncover novel PPARα-regulated genes and pathways in liver.

## 1. INTRODUCTION

The peroxisome-proliferator-activated receptors (PPARs) play a pivotal role in the regulation of nutrient metabolism. PPARs are ligand-activated transcription factors that belong to the superfamily of nuclear hormone receptors [1–3]. They share a common mode of action that involves formation of heterodimers with the nuclear receptor RXR, followed by binding to specific DNA-response elements in the promoter of target genes. The genomic sequence recognized by PPARs, referred to as PPAR response element or PPRE, consists of a direct repeat of the consensus hexameric motif AGGTCA interspaced by a single nucleotide. Binding of ligands to PPARs leads to recruitment of coactivators and causes chromatin remodeling, resulting in initiation of DNA transcription and upregulation of specific PPAR target genes [4, 5]. Ligands for PPARs include both endogenous compounds, such as fatty acids and their eicosanoid derivatives, and synthetic agonists. Three different PPAR subtypes have been identified: PPARα, PPARβ/δ, and PPARγ. The latter isotype, which is most highly expressed in adipose tissue, is known to play an important role in adipocyte differentiation and lipid storage [6–8]. It is a target for an important class of antidiabetic drugs, the insulin-sensitizing thiazolidinediones. Expression of PPARβ/δ is ubiquitous and has been connected to wound healing, cholesterol metabolism, and fatty acid oxidation in adipose tissue and muscle [9–12]. Finally, PPARα is highly expressed in liver where it stimulates fatty acid uptake and activation, mitochondrial β-oxidation, peroxisomal fatty acid oxidation, ketogenesis, and fatty acid elongation and desaturation. In addition, it has a major role in glucose metabolism [13] and the hepatic acute phase response [14, 15]. Importantly, PPARα is the molecular target for the hypolipidemic fibrate class of drugs that lower plasma triglycerides and elevate plasma HDL (high-density lipoprotein) levels.

In recent years, microarray technology has emerged as a powerful technique to study global gene expression. In theory, microarray analysis is a terrific tool to map PPARα-dependent genes and further characterizes PPARα function. In practice, microarray yields a huge amount of data, the analysis and interpretation of which can be very difficult. Numerous studies have examined the effect of synthetic PPARα agonists on global gene expression using microarrays. While these studies uncovered many possible PPARα target genes, the manner in which the data were presented often rendered interpretation difficult. Part of the complexity is due to the size of the PPARα-dependent transcriptome in liver, which easily exceeds one thousand genes.

The aim of the present study was twofold: (1) to generate a comprehensive overview of PPARα-regulated genes relevant to hepatic lipid metabolism and (2) to identify possible novel target genes and target pathways of PPARα connected with lipid metabolism. To that end, we (1) combined microarray data from several independent animal experiments involving PPARα-null mice (in these experiments, mice were either given Wy14643 or fasted for 24 hours), (2) focused on up-regulation of genes by PPARα in conformity with the general paradigm of transcriptional regulation by nuclear hormone receptors, and (3) reduced complexity by progressively moving from the complete PPARα-dependent transcriptome towards genes relevant to lipid metabolism, and finally to the identification of possible PPARα target genes involved in lipid metabolism.

## 2. METHODS AND MATERIALS

### 2.1. Materials

Wy14643 was obtained from ChemSyn Laboratories (Lenexa, KS). Recombinant human insulin (Actrapid) was from Novo Nordisk (Copenhagen, Denmark). SYBR Green was from Eurogentec (Seraing, Belgium). DMEM, fetal calf serum, calf serum, and penicillin/streptomycin/fungizone were from Lonza Bioscience (Verviers, Belgium). Otherwise, chemicals were from Sigma (Zwijndrecht, The Netherlands).

### 2.2. Animals

Male pure-bred Sv129 and PPARα-null mice on a Sv129 background were used at 3–5 months of age (Jackson Laboratories, Bar Harbor, ME). Animals were fed normal laboratory chow (RMH-B diet, Arie Blok animal feed, Woerden, the Netherlands). Study 1: fed mice were killed at the end of the dark cycle. Fasting was started at the onset of the light cycle for 24 hours (n=5 per group). Studies 2 and 4: wild-type and PPARα-null mice were fed with Wy14643 for 5 days by mixing it in their food (0.1%, n=5 per group). Studies 2 and 4 were carried out independently and 2 years apart. Study 3: wild-type and PPARα-null mice fasted for 4 hours received a single dose of Wy14643 (400 μL of 10 mg/mL Wy14643 dissolved in 0.5% carboxymethylcellulose) and were killed 6 hours later (n=5 per group). Study 5: wild-type and PPARα-null mice at 2-3 months of age were given a high-fat diet (D12451, Research Diets, New Brunswick, NJ) for 20 weeks (composition available at http://www.researchdiets.com/pdf/Data%20Sheets/DIO%20Series.pdf). During the last week, half of the mice were given Wy14643 for 7 days by mixing it in their food (0.1%, n=5 per group). Livers were dissected and immediately frozen in liquid nitrogen.

All animal experiments were approved by the animal experimentation committee of Wageningen University and were carried out in conformity with the public health service (PHS) policy on humane care and use of laboratory animals.

### 2.3. Primary hepatocytes

Rat (Wistar) and mouse (sv129) hepatocytes were isolated by two-step collagenase perfusion as described previously [16]. Cells were plated on collagen-coated six-well plates. Viability was determined by Trypan Blue exclusion, and was at least 75%. Hepatocytes were suspended in William's E medium (Lonza Bioscience, Verviers, Belgium) supplemented with 10% (v/v) foetal calf serum, 20 m-units/mL insulin, 50 nM dexamethasone, 100 U/mL penicillin, 100 μg/mL of streptomycin, 0.25 μg/mL fungizone, and 50 μg/mL gentamycin. The next day, cells were incubated in fresh medium in the presence or absence of Wy14643 (10 μM) dissolved in DMSO for 24 hours, followed by RNA isolation.

Human hepatocytes and Hepatocyte Culture Medium Bulletkit were purchased from Lonza Bioscience (Verviers, Belgium). Human hepatocytes were isolated from a single donor. Cells were plated on collagen-coated six-well plates. Upon arrival of the cells, the medium was discarded and was replaced by Hepatocyte Culture Medium. The next day, cells were incubated in fresh medium in the presence or absence of Wy14643 (50 μM) dissolved in DMSO for 12 hours, followed by RNA isolation.

### 2.4. Affymetrix microarray

Total RNA was prepared from mouse livers and primary hepatocytes using TRIzol reagent (Invitrogen, Breda, The Netherlands). RNA was either pooled per group or treatment (studies 1 and 2, primary hepatocytes), or used individually (studies 3 and 4), and further purified using RNeasy micro columns (Qiagen, Venlo, the Netherlands). RNA integrity was checked on an Agilent 2100 bioanalyzer (Agilent Technologies, Amsterdam, the Netherlands) using 6000 Nano Chips according to the manufacturer's instructions. RNA was judged as suitable for array hybridization only if samples exhibited intact bands corresponding to the 18S and 28S ribosomal RNA subunits, and displayed no chromosomal peaks or RNA degradation products (RNA Integrity Number > 8.0). Ten micrograms of RNA were used for one cycle cRNA synthesis (Affymetrix, Santa Clara, CA). Hybridization, washing, and scanning of Affymetrix Genechip MOE430 (studies 1 and 2) or mouse genome 430 2.0 arrays (studies 3 and 4) was according to standard Affymetrix protocols.

Scans of the Affymetrix arrays were processed using packages from the Bioconductor project [17]. Expression levels of probe sets were calculated using GCRMA [18], followed by identification of differentially expressed probe sets using Limma [19]. Comparison was between fasted wild-type and fasted PPARα-null mice (study 1) or between Wy14643-treated wild-type and Wy14643-treated PPARα-null mice (studies 2–4). P-values were corrected for multiple testing using a false discovery rate method [20]. Probe sets that satisfied the criterion of FDR < 1% (q-value <0.01) and fold-change >1.5 were considered to be significantly regulated. Functional clustering of the array data was performed by a method based on overrepresentation of Gene Ontology (GO) terms [21].

For the primary hepatocytes, expression levels were calculated applying the multichip-modified gamma model for oligonucleotide signal (multi-mgMOS) [22] and a remapped chip description file [23].

All microarray datasets were deposited to gene expression omnibus (GEO). The GEO series accession numbers are as follows: study 1: GSE8290, study 2: GSE8291, study 3: GES 8292, study 4: GSE8295, primary hepatocytes: GSE8302.

### 2.5. RNA isolation and Q-PCR

Total RNA was extracted from tissues with TRIzol reagent (Invitrogen, Breda, the Netherlands). 1 μg of total RNA was reverse-transcribed with iScript (Bio-Rad, Veenendaal, the Netherlands). cDNA was PCR-amplified with Platinum Taq DNA polymerase (Invitrogen) on a Bio-Rad iCycler or MyIQ PCR machine. Primers were designed to generate a PCR amplification product of 100–200 bp and were taken from Primerbank (http://pga.mgh.harvard.edu/primerbank). Specificity of the amplification was verified by melt-curve analysis and evaluation of efficiency of PCR amplification. The sequence of primers used is available upon request. The mRNA expression of all genes reported was normalized to 36B4 or cyclophilin gene expression.

### 2.6. In silico screening of putative PPREs using a PPRE classifier

Genomic sequences for mouse genes spanning 20 kbp centered at the transcriptional start site (TSS) were extracted from the Ensembl database (NBCI36) and screened for DR1-type REs with predicted binding strength of at least 1%. The binding strength prediction was based on a PPRE classifier that uses a database of in vitro binding data for PPARs to assign predicted binding strength according to a classification scheme (Matilainen et al. submitted). The conservation of the putative PPREs between mouse, human, dog, and rat were evaluated using the Vertebrate Multiz Alignment and Conservation track available from UCSC genome browser (NCBI releases for human and mouse genomes, hg18 and mm8, February 2006).

### 2.7. Histological examination of liver

5 μ sections were cut from frozen liver pieces. For oil red O staining, sections were air dried for 30 minutes, followed by fixation in formal calcium (4% formaldehyde, 1% CaCl_2_). Oil red O stock solution was prepared by dissolving 0.5 g oil red O in 500 mL isopropanol. An oil red O working solution was prepared by mixing 30 mL oil red O stock with 20 mL dH_2_O. Sections were immersed on working solution for 10 minutes followed by extensive washes in H_2_O. Haematoxylin and eosin staining of frozen liver sections was carried out as described (http://www.ihcworld.com/histology.htm).

## 3. RESULTS

### 3.1. Global analysis of PPARα-dependent gene regulation

**Figure 1:**
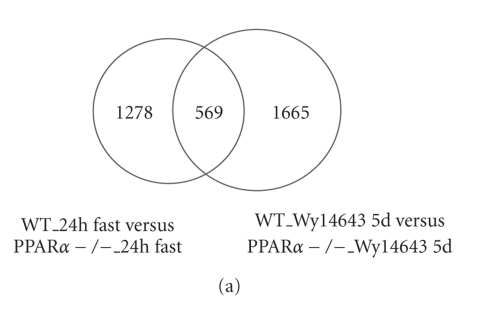

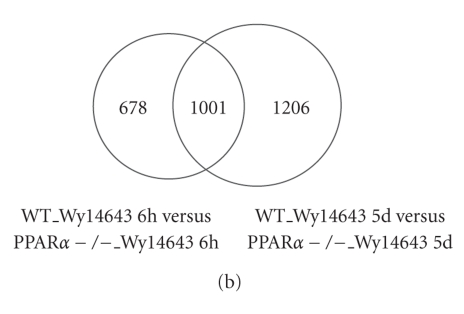
Microarray analysis of PPARα-dependent gene regulation in mouse liver. (a) Venn diagram showing the number of differentially expressed probesets between livers of 24-hour fasted wild-type and PPARα-null mice, and between wild-type and PPARα-null mice treated with the PPARα agonist Wy14643 for 5 days. Pooled RNA was hybridized to Affymetrix MOE430A arrays. A fold-change of >1.5 was used as cutoff. (b) Venn diagram showing the number of differentially expressed probesets between livers of wild-type and PPARα-null mice treated with the PPARα agonist Wy14643 for 6 hours, and between wild-type and PPARα-null mice treated with the PPARα agonist Wy14643 for 5 days. RNA from individual mice was hybridized to mouse 430 2.0 arrays. Probesets that satisfied the criteria of fold-change >1.5 and FDR <0.01 were considered to be significantly regulated.

We analyzed the data from 4 independent microarray studies to obtain a comprehensive picture of PPARα-dependent upregulation of gene expression in mouse liver. In the first study, mRNA was compared between livers of 24-hour fasted wild-type and PPARα-null mice. In the second study, mRNA was compared between liver of wild-type mice and PPARα-null mice fed the PPARα agonist Wy14643 for 5 days. In these two studies, RNA was pooled from 4-5 mice and hybridized to Affymetrix MOE430A GeneChip arrays. Since no biological replicates were analyzed, only a fold-change threshold criteria could be applied. Using a cutoff of 1.5-fold, expression of a total of 1847 probesets was lower in 24-hour fasted PPARα-null mice compared with 24-hour fasted wild-type mice (Figure 1(a)) (http://nutrigene.4t.com/microarray/ppar2007). Using the same cutoff, 2234 probesets were at least 1.5-fold lower in the livers of PPARα-null mice fed Wy14643 compared to wild-type mice fed Wy14643 (http://nutrigene.4t.com/microarray/ppar2007). The number of probesets that overlapped between the two groups was 569. A large proportion of these genes, which are thus under control of PPARα under pharmacological and physiological conditions, may represent target genes of PPARα.

In the third study, mRNA was compared between livers of wild-type mice and PPARα-null mice treated with Wy14643 for 6 hours, while in the fourth study mRNA was compared between livers of wild-type mice and PPARα-null mice fed Wy14643 for 5 days. Study 4 was carried out independently of study 2 in a different set of mice. For these two studies, biological replicates (4-5 mice per group) were run using Affymetrix mouse genome 430 2.0 GeneChip array, enabling statistical analysis of the data which was not possible for studies 1 and 2. Applying a false discovery rate of 0.01 and a 1.5-fold cutoff, 1679 probesets were lower in the livers of PPARα-null mice compared to wild-type mice 6 hours after treatment with Wy14643, and 2207 probesets after 5 days of feeding Wy14643 (Figure 1(b)) (http://nutrigene.4t.com/microarray/ppar2007). While the majority of genes regulated by PPARα after 6 hours of Wy14643 treatment were also, and generally more significantly, regulated after 5 days of Wy14643 treatment (overlap of 1001 probesets), many genes were specifically or more significantly regulated after 6 hours, including the direct PPAR target G0S2 and the EL gene, respectively. The complete set of data from studies 2 and 4, which includes up- and downregulated genes, has been submitted to the Peroxisome Proliferators compendium assembled by Dr. J.C. Corton (US EPA, Research Triangle Park, USA). They will be analyzed in conjunction with numerous other microarray experiments involving peroxisome proliferators to obtain the “peroxisome proliferator transcriptome.” In addition, the datasets have been submitted to GEO.

### 3.2. Pathway analysis of PPARα-dependent gene regulation

Functional clustering analysis of the microarray data by Gene Ontology classification indicated that numerous Gene Ontology classes were overrepresented among the genes that were >1.5-fold upregulated in 24-hour fasted wild-type compared to 24-hour fasted PPARα-null mice. The same was true for the comparison between wild-type and PPARα-null mice treated with Wy14643 for 5 days. Among the overrepresented Gene Ontology classes, we found many classes that are known to be governed by PPARα, including fatty acid beta-oxidation, acyl-CoA metabolism, leukotriene metabolism, and peroxisome organization and biogenesis (http://nutrigene.4t.com/microarray/ppar2007). Interestingly, we also noticed that numerous Gene Ontology classes were specifically upregulated by PPARα under fasting conditions or by Wy14643 feeding. The data suggest, for example, that pyruvate metabolism and posttranslational protein targeting to membrane are specifically regulated in a PPARα-dependent manner by Wy14643 but not by fasting. Indeed, it is clear that some genes (e.g., Acot2 and Cd36) are PPARα-dependently regulated by Wy14643 and much less so by fasting, whereas others (e.g., Gpam, Hmgcs2) are PPARα dependently regulated by fasting and much less so by Wy14643. However, it is important to emphasize that the ErmineJ Gene Ontology classification, as any functional clustering analysis, needs to be interpreted carefully.

The Gene Ontology classification analysis of the comparison wild-type versus PPARα-null mice treated with Wy14643 for 6 hours (study 3) was almost identical to the analysis for mice treated with Wy14643 for 5 days (study 4), suggesting that most of the gene expression changes elicited by Wy14643 treatment are fast transcriptional responses in correspondence with direct regulation of gene expression by PPARα. One notable exception was the class representing the acute phase response, which was regulated by 5-day but not 6-hour treatment with Wy14643.

### 3.3. Comprehensive list of PPARα-targets involved in lipid metabolism

**Figure 2:**
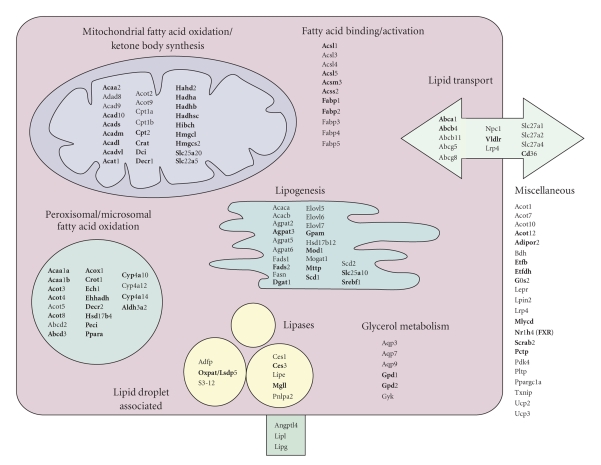
Overview of PPARα-regulated genes involved in hepatic lipid metabolism. Genes in bold are PPARα-dependently regulated during fasting and by Wy14643, representing a conservative list of PPARα targets. Genes in normal font are PPARα dependently regulated in any of the four studies included. Functional classification is based on a self-made functional annotation system of genes involved in lipid metabolism (http://nutrigene.4t.com/microarray/ppar2007).

Using these lists of genes that are upregulated by PPARα in mouse liver, we were able to create a comprehensive picture of PPARα-regulated genes connected with lipid metabolism. Genes in bold are PPARα dependently regulated by Wy14643 and during fasting, representing a conservative list of PPARα targets (Figure 2). Genes in normal font are PPARα dependently regulated in any of the four studies included. From this picture, it is evident that rather than merely regulating the rate limiting enzyme in fatty acid oxidation, PPARα appears to regulate virtually every single step in the peroxisomal and mitochondrial fatty acid oxidation pathway. Furthermore, many genes involved in fatty acid binding and activation, lipid transport, and glycerol metabolism were controlled by PPARα. What is remarkable is that PPARα also governs the expression of numerous genes involved in the synthesis of fats, which runs counter to the idea that PPARα mainly regulates fat catabolism. Several genes belonging to the lipogenic pathway have previously been recognized as PPARα targets, including Mod1 and Scd1, yet the extent of regulation by PPARα is unexpected [24]. Regulation of lipogenesis by PPARα was mainly observed after Wy14643 treatment, and to a much lesser extent after fasting.

### 3.4. Novel putative targets of PPARα involved in lipid metabolism

**Figure 3:**
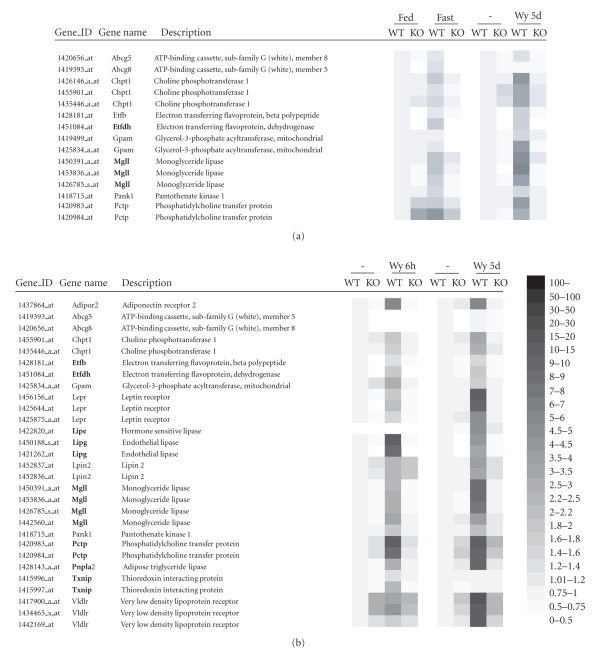
PPARα-dependent regulation in mouse liver of selected genes involved in lipid metabolism as shown by heat map. The (GCRMA normalized) expression data were derived from 4 separate microarray studies. Expression levels in wild-type mice without treatment were set at 1. (a) Expression data derived from studies 1 and 2. (b) Expression data derived from studies 3 and 4. Genes in bold were selected for expression analysis by Q-PCR and in silico screening for putative PPREs.

In addition to providing an overview of PPARα-dependent gene regulation, we were interested in identifying novel PPARα-regulated genes that are implicated in lipid metabolism. To that end, we went through the array data from studies 1 and 2 on the one hand, and studies 3 and 4 on the other hand, and selected a number of genes to generate a heat map showing their PPARα-dependent upregulation by fasting and/or Wy14643 (Figure 3). To our knowledge, none of the genes shown, all of which are involved in hepatic lipid metabolism, has yet been reported to be regulated by PPARα. This includes phosphatidylcholine transfer protein (lipoprotein metabolism), glycerol-3-phosphate acyltransferase (triglyceride synthesis), very low-density lipoprotein receptor, choline phosphotransferase (phosphatidylcholine synthesis), and leptin receptor. Since all of these genes, except Abcg5, Abcg8, and Lipe, were upregulated 6 hours after Wy14643 treatment, they possibly represent novel direct target genes of PPARα in liver, although PPREs have yet to be identified in their respective gene promoters.

**Figure 4:**
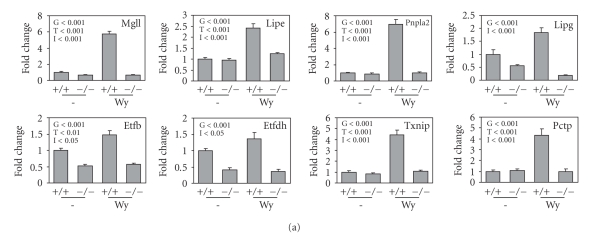

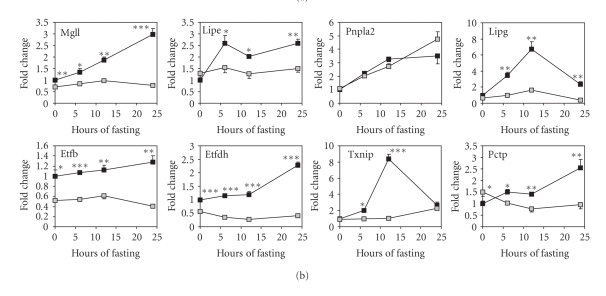
PPARα governs expression of selected genes in mouse liver. (a) Regulation of expression of selected genes by Wy14643-feeding (5 days) in liver of wild-type (+/+) and PPARα-null mice (−/−), as determined by Q-PCR. Error bars represent SEM. Differences were evaluated statistically using two-way ANOVA. Significance (p-value) of effect of genotype (G), treatment (T) and interaction (I) between genotype and treatment is indicated in each figure. (b) Regulation of expression of selected genes by fasting in liver of wild-type (▪) and PPARα-null mice (□), as determined by Q-PCR. Error bars represent SEM. Differences in expression between wild-type and PPARα-null mice at each time point were evaluated by Student t test. *P<.05; **P<.01; ***P<.001.

Eight genes (shown in bold, Figure 3) were selected for more detailed investigation of PPARα-dependent gene regulation. Three of these genes are expected to be involved in the breakdown of hepatic triglycerides towards fatty acids: adipose triglyceride lipase (Pnpla2), hormone sensitive lipase (Lipe), and monoglyceride lipase (Mgll). Recent studies suggest that this threesome of genes is responsible for adipose tissue lipolysis [25–27]. In addition, we selected endothelial lipase (EL, Lipg), a recently identified member of triglyceride lipase gene family that is a major determinant of plasma HDL cholesterol [28–30], and electron transferring flavoprotein dehydrogenase (Etfdh) and electron transferring flavoprotein β polypeptide (Etfb), which are components of the electron transport chain and accept electrons from at least nine mitochondrial matrix flavoprotein dehydrogenases [31, 32]. Finally, we selected phosphatidylcholine transfer protein (Pctp), which is involved in lipoprotein metabolism, and thioredoxin interacting protein (Txnip), which was recently identified as a major regulator of the hepatic response to fasting, similar to PPARα. The selection of these genes was based entirely on perceived novelty and potential functional importance of the observed regulation. Using real-time quantitative PCR (Q-PCR), we confirmed that the expression of all 8 genes in liver was increased by Wy14643 feeding in a PPARα-dependent manner (Figure 4(a)). In addition, we measured regulation of expression of this set of genes by PPARα during the course of fasting (Figure 4(b)). Expression of all 8 genes went up during fasting which, except for Pnpla2, was PPARα-dependent. However, the pattern of expression was remarkably different between the various genes, suggesting for each gene a complex and unique interplay between several fasting-dependent transcription factors, including PPARα.

### 3.5. PPARα-dependent regulation in primary hepatocytes

**Figure 5:**
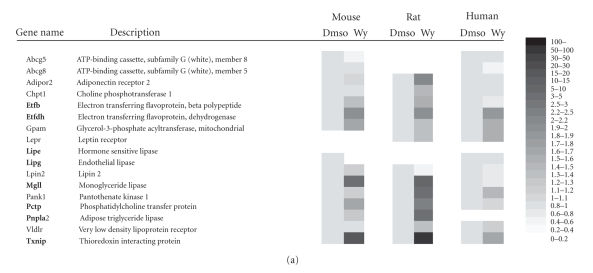

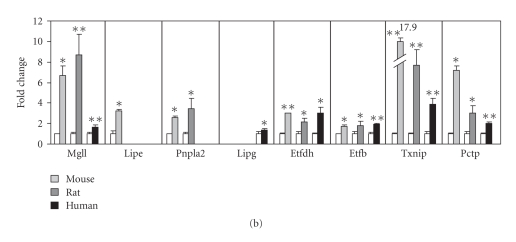
Regulation of selected genes involved in lipid metabolism in primary hepatocytes by Wy14643. (a) Microarray-based heat map showing relative expression levels of genes calculated using a multichip modified gamma model for oligonucleotide signal (multi-mgMOS) and a remapped chip description file. Expression levels in the absence of ligand were set at 1. (b) Relative induction of expression of selected genes in primary hepatocytes by Wy14643, as determined by Q-PCR. The primary hepatocytes used for Q-PCR and microarray analysis were from independent experiments. Genes were not included when expression was extremely low (Ct > 30). Error bars represent SD. The effect of Wy14643 on gene expression was evaluated by Student t test. *P<.05; **P<.01.

To examine whether the PPARα-dependent regulation of the set of genes shown in Figure 3 was not an indirect consequence of metabolic perturbations elicited by the experimental challenge, we studied the effect of PPARα activation in primary mouse, rat, and human hepatocytes. Gene expression was first analyzed by microarray (Figure 5(a)), followed by targeted analysis of the selected 8 genes by Q-PCR (Figure 5(b)). Expression levels were calculated by applying a multichip modified gamma model for oligonucleotide signal (multi-mgMOS) [22] and a remapped chip description file [23] to allow for parallel analysis of the same gene within different species. Expression of almost every gene studied was highly upregulated by Wy14643 in mouse and rat hepatocytes, compared to a more modest or no induction in human hepatocytes. For reasons that are not completely clear, in human hepatocytes, data from Q-PCR and microarray did not always perfectly align. Overall, the data indicate that the PPARα-dependent regulation observed in vivo can be reproduced in primary hepatocytes. Furthermore, the data suggest that expression of 6 genes is governed by PPARα in human as well.

### 3.6. In silico screening of putative PPREs

**Figure 6:**
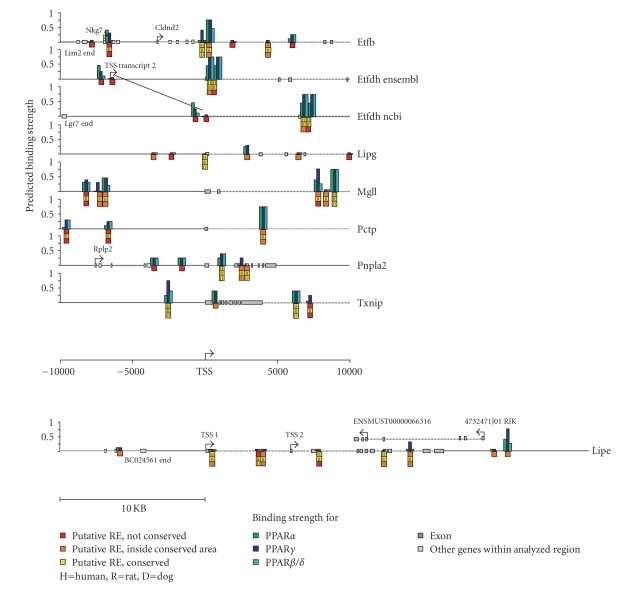
In silico screening for putative PPREs for the selected 8 genes, 10 kbp up- and downstream of the transcriptional start site were examined for the presence of putative PPREs. For each putative PPRE identified, the predicted PPAR subtype specific binding strength was determined, as reflected by the height of the bar. The sequence conservation of the PPRE among various species is indicated.

To evaluate whether the selected eight genes represent possible direct PPAR target genes, the (mouse) genes were analyzed for the presence of putative PPREs using an in silico screening method (Figure 6). Ten kbp up- and downstream of the TSS were examined. For each putative PPRE identified, the predicted PPAR subtype specific binding strength was determined. For each gene, at least one PPRE was identified that was conserved among rat, dog, and human. The Etfdh and Txnip genes were characterized by the presence of two very strong putative PPREs that were conserved in human. Up to six putative PPREs were identified in the Mgll gene, only one of which was conserved in human. A similar picture was found for Pnpla2. The putative PPREs located in the EL gene were weak and generally not conserved. Interestingly, a strong putative PPRE was identified in the Pctp gene, which however was not conserved in human. Conversely, the human Pctp gene contained several putative PPREs that were not conserved in mouse (data not shown).

### 3.7. PPARα activation prevents hepatic lipid storage after fasting

**Figure 7:**
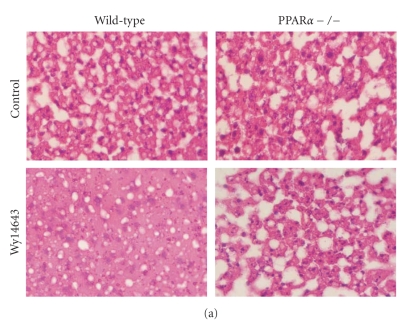

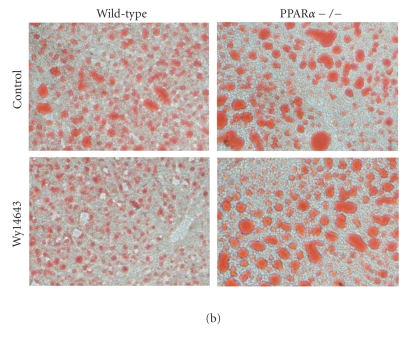

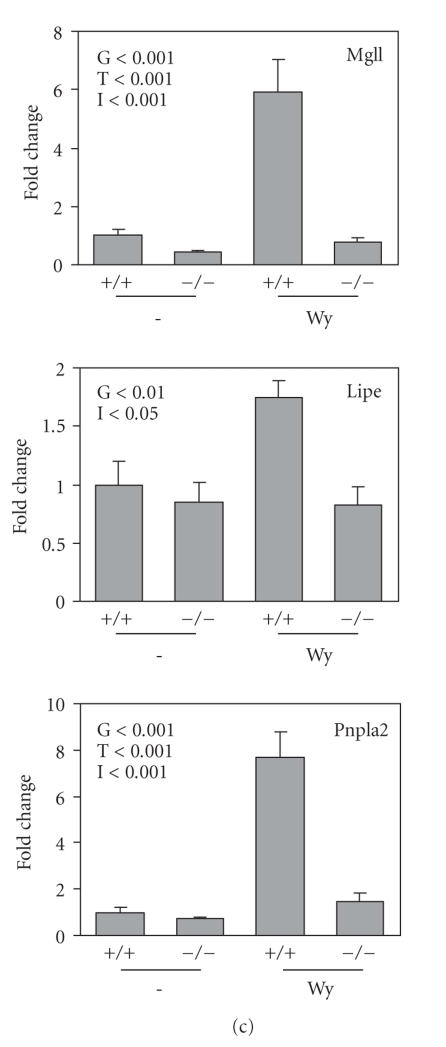
Induction of the triglyceride hydrolysis pathway by Wy14643 is paralleled by a decrease in hepatic lipid stores. Hematoxilin and eosin staining (a) and oil red O staining (b) of representative liver sections of wild-type and PPARα-null mice treated or not with Wy14643 for 7 days (magnification 200X). All mice were given an HFD for 20 weeks prior to Wy14643 treatment. (c) Hepatic expression of Mgll, Lipe, and Pnpla2 in the 4 experimental groups as determined by Q-PCR. Error bars represent SEM. Differences were evaluated statistically using two-way ANOVA. Significance (p-value) of effect of genotype (G), treatment (T), and interaction (I) between genotype and treatment is indicated in each figure.

Our data extend the role of PPARα in hepatic lipid metabolism and suggest that PPARα may govern triglyceride hydrolysis. To find out whether activation of the triglyceride hydrolysis pathway by PPARα is associated with a decrease in hepatic triglyceride stores, we compared wild-type and PPARα-null mice fed an HFD for 20 weeks, followed by treatment for one week with Wy14643. Numerous studies, including ours [33], have shown that chronic HFD increases hepatic triglyceride stores. In wild-type mice fed the HFD, treatment with Wy14643 markedly decreased hepatic lipids (Figures 7(a) and 7(b)), as shown by smaller lipid droplets, which was paralleled by significant induction of expression of Pnpla2, Lipe, and Mgll (Figure 7(c)). These data suggest that induction of the triglyceride hydrolysis pathway may contribute to the overall reduction in liver triglycerides elicited by PPARα activation.

## 4. DISCUSSION

The aim of our study was twofold: (1) to generate a comprehensive overview of PPARα-regulated genes relevant to hepatic lipid metabolism, and (2) to identify possible novel target genes and target pathways of PPARα connected with lipid metabolism.

It can be argued that to identify possible novel PPARα targets, the proper comparison should have been between wild-type and wild-type treated with Wy14643, as opposed to wild-type treated with Wy14643 and PPARα-null treated with Wy14643, in order to avoid inclusion of genes that are differentially expressed between wild-type and PPARα-null mice under basal conditions (and could represent genes indirectly regulated by PPARα). The rationale behind our decision was that we wanted to be open-minded about the PPARα-dependent transcriptome and not exclude genes that are solely regulated by PPARα under basal conditions. For example, opting for the comparison wild-type versus wild-type treated with Wy14643 would have led to the exclusion of Etfdh, which according to our data represents a prime candidate PPARα target gene in mouse and human. Furthermore, to enable comparison between the effects of fasting and Wy14643, it was essential to include the PPARα dependency, since the majority of genes regulated by fasting are regulated in a PPARα-independent manner.

Gene Ontology classification analysis showed that numerous pathways and biological processes beyond lipid metabolism were regulated by PPARα. We observed that the expression of almost 1700 probesets was significantly increased 6 hours after a single oral dose of Wy14643. Although not all genes regulated may represent direct PPARα targets, and even though the functional consequences of the observed regulation still needs to be demonstrated, these data at least suggest a major role for PPARα in hepatic gene expression and overall liver homeostasis.

 In agreement with the first aim, we created a comprehensive overview of hepatic PPARα-regulated genes connected to lipid metabolism (Figure 2). A functional PPRE has been found in the promoter of several of these genes, classifying them as direct PPARα target genes, and many more genes have been shown to be upregulated by PPARα without a functional PPRE having been identified [24]. It can be presumed that the far majority of genes presented in Figure 2 (as well as the other genes that were shown to be regulated by PPARα) are actually direct target genes of PPARα, but it is beyond the scope and capacity of the present study to address this issue in more detail. Our hope is that by combination of expression arrays with global analysis of promoter occupancy by PPARα using chromatin immunoprecipitation and tiling or promoter arrays (so-called ChIP-on Chip analysis), the complete picture of direct PPARα target genes will be available in the future.

The second aim of our study was to identify possible novel target genes of PPARα representing specific steps in lipid metabolism unknown to be governed by PPARα. As part of this effort, we identified several genes for which a link with PPARα has not yet been reported, including VLDL receptor, leptin receptor, and choline phosphotransferase. We focused our energy on 8 genes for which regulation by PPARα was deemed most novel and functionally interesting. All 8 genes, except for Lipe, were significantly upregulated 6 hours after treatment with Wy14643.

Using an in silico method to screen for PPREs, for each gene several putative PPREs could be located within 10 kbp of the transcriptional start site. Within this region, at least one PPRE was identified that was conserved among rat, dog, and human. The presence of multiple strong putative PPREs within the mouse Mgll gene is in correspondence with the marked regulation of Mgll expression in mouse liver and isolated hepatocytes. To a lesser extent, this is also true for the Pnpla2 and Pctp genes. Furthermore, the predicted presence of 2 strong, well-conserved putative PPREs in the Etfdh and Txnip genes is in agreement with the highest fold-induction of these genes by Wy14643 in primary human hepatocytes. Although in silico screening may not be able to substitute for analysis of direct promoter binding by ChIP, the predictive power of the method explored has been shown to be remarkably robust (Matilainen et al. submitted). Our results also substantiate the developing notion that PPAR-dependent gene regulation is generally mediated by multiple PPREs, rather than a single PPRE.

One remarkable outcome of the global analysis of gene regulation by PPARα is that PPARα appears to play a major role in governing lipogenesis. While several genes involved in lipogenesis were already known as PPARα targets, including Δ5 and Δ5 desaturase (Fads), stearoyl-CoA desaturase (Scd), microsomal triglyceride transfer protein (Mttp), and malic enzyme (Mod1) [24], the extent of regulation of lipogenesis is somewhat surprising, especially since PPARα is generally considered to stimulate fat catabolism rather than fat synthesis. It can be speculated that upregulation of fatty acid desaturation and elongation enzymes by PPARα might serve to stimulate production of PPARα ligands, and is part of a feed-forward action of PPARα that also includes autoregulation of gene expression.

Although the triglyceride hydrolysis pathway in liver still has to be fully elucidated, it may very well be similar to the pathway operating in adipose tissue [27]. Adipose tissue triglycerides are likely hydrolyzed in a three-step process catalyzed by adipose triglyceride lipase (Pnpla2), hormone sensitive lipase (Lipe), and monoglyceride lipase (Mgll) [25–27, 34]. Remarkably, deletion of the Pnpla2 gene in mice not only results in more adipose mass but also causes a marked increase in lipid storage in a variety of organs, including liver and heart, suggesting that the triglyceride hydrolysis pathway is conserved between various organs [27]. Disabling the PPARα gene is known to increase hepatic triglyceride accumulation, especially under conditions of fasting [33, 35, 36]. Conversely, treatment with PPARα agonists lowers hepatic triglyceride levels in various models of hepatic steatosis [37–40]. The antisteatotic effect of PPARα has generally been ascribed to stimulation of fatty acid oxidation, which, by decreasing intracellular fatty acid levels, will act as a drain on intracellular triglyceride stores. However, our data suggest that PPARα may directly govern the triglyceride hydrolysis pathway in liver via upregulation of lipases Pnpla2, Lipe, Mgll, and possibly Ces1 and Ces3 (Figure 2). Although it is impossible to provide definite experimental proof that induction of the triglyceride hydrolysis pathway by PPARα, or induction of fatty acid oxidation for that matter, is necessary and sufficient for its hepatic triglyceride-lowering effect, it likely contributes to the overall reduction in liver triglycerides elicited by PPARα agonists.

Our data suggest that expression of EL is under control of PPARα. EL, synthesized in endothelial cells, plays an important role in governing plasma lipoprotein concentrations and is a major determinant of plasma HDL cholesterol and apoAI concentrations. Indeed, overexpression of EL in the liver results in a significant decrease in HDL cholesterol and apoAI [28–30]. EL has been shown to have some triglyceride lipase but mainly phospolipase activity [41]. Although in silico screening failed to detect a strong PPREs in this gene, in our study EL expression was highly increased by 6 hours Wy14643 treatment and by fasting in a PPARα-dependent manner, suggesting that EL may be a direct PPARα target gene. As EL expression was minimal in primary hepatocytes, EL transcripts likely originated from liver epithelial cells rather than liver parenchymal cells. Although further work is necessary, we suspect that EL may be a direct PPARα target in endothelial cells. Considering that, in contrast to EL, PPARα agonists raise plasma HDL, the functional importance of regulation of EL by PPARα needs to be further validated.

Another novel PPARα-regulated gene of relevance to lipoprotein metabolism is Pctp. Pctp is a steroidogenic acute regulatory-related transfer domain protein that binds phosphatidylcholines with high specificity. Studies with Pctp-null mice suggest that it may modulate HDL particle size and rates of hepatic clearance [42]. According to our data, expression of Pctp increases during fasting, which is abolished in PPARα-null mice. Wy14643 markedly upregulated Pctp mRNA in mouse liver as well as in mouse, rat, and human hepatocytes, suggesting it may represent a novel PPARα target gene.

Etfdh and Etfb are essential components of the oxidative phosphorylation pathway. They are responsible for the electron transfer from at least 9 mitochondrial flavin-containing dehydrogenases to the main respiratory chain [31, 32]. According to our data, expression of Etfdh and Etfb is governed by PPARα, suggesting that besides the β-oxidation pathway, PPARα also regulates components of the respiratory chain involved in the transfer of electrons from fatty acids and other molecules.

The last gene that we studied in more detail was Txnip, which is also known as Hyplip1. A spontaneous mutation within the Txnip gene gives rise to a complex phenotype that resembles familial-combined hyperlipidemia, including hypercholesterolemia and hypertriglyceridemia [43]. Recent studies suggest that Txnip plays an important metabolic role in the fasting-feeding transition by altering the redox status of the cell, which results in stimulation of the tricarboxylic acid cycle at the expense of ketone body or fatty acid synthesis [44]. Indeed, Txnip-deficient mice show elevated plasma ketones, elevated free fatty acids, hypercholesterolemia, and hypertriglyceridemia, yet decreased glucose levels [43, 45]. The phenotype is very similar to that of PPARα-null mice with the exception of the elevated plasma ketones. Since hepatic expression of Txnip is decreased in PPARα-null mice, it can be hypothesized that part of the effect of PPARα deletion on lipid and glucose metabolism is mediated by downregulation of Txnip in liver, which subsequently might affect redox status. It is unclear to what extent Txnip expression is affected by PPARα deletion in tissues other than liver.

In conclusion, our data indicate that the role of PPARα in hepatic lipid metabolism is much more extensive than previously envisioned. By generating a schematic overview of PPARα-dependent gene regulation in mouse liver, and, for a selected set of genes, by providing evidence for direct regulation by PPARα in rodents and human, we have extended the role of PPARα in the control of hepatic lipid metabolism.
